# The Role of Adenosine in Pulmonary Vein Isolation: A Critical Review

**DOI:** 10.1155/2016/8632509

**Published:** 2016-02-15

**Authors:** Paolo D. Dallaglio, Timothy R. Betts, Matthew Ginks, Yaver Bashir, Ignasi Anguera, Kim Rajappan

**Affiliations:** ^1^Heart Disease Institute, Bellvitge Biomedical Research Institute (IDIBELL), Bellvitge University Hospital, 08907 Barcelona, Spain; ^2^Electrophysiology Department, Oxford Heart Centre, John Radcliffe Hospital, Oxford University Hospitals, Oxford OX39DU, UK

## Abstract

The cornerstone of atrial fibrillation (AF) ablation is pulmonary vein isolation (PVI), which can be achieved in more than 95% of patients at the end of the procedure. However, AF recurrence rates remain high and are related to recovery of PV conduction. Adenosine testing is used to unmask dormant pulmonary vein conduction (DC). The aim of this study is to review the available literature addressing the role of adenosine testing and determine the impact of ablation at sites of PV reconnection on freedom from AF. Adenosine infusion, by restoring the excitability threshold, unmasks reversible injury that could lead to recovery of PV conduction. The studies included in this review suggest that adenosine is useful to unmask nontransmural lesions at risk of reconnection and that further ablation at sites of DC is associated with improvement in freedom from AF. Nevertheless it has been demonstrated that adenosine is not able to predict all veins at risk of later reconnection, which means that veins without DC are not necessarily at low risk. The role of the waiting period in the setting of adenosine testing has also been analyzed, suggesting that in the acute phase adenosine use should be accompanied by enough waiting time.

## 1. Introduction

Catheter ablation is an effective treatment for atrial fibrillation (AF); the cornerstone of this procedure is durable and effective pulmonary vein isolation (PVI). This is particularly important for paroxysmal AF, whose pathophysiological basis resides in the electrical properties of the strands of muscular tissue that propagate from the left atrium (LA) into the pulmonary veins [1]. These “transition” zones show anisotropic conduction and can have altered excitability that may be able to initiate AF. Following the recommendation of the Heart Rhythm Society and the European Heart Rhythm Association Guidelines in many centers, the first step of AF ablation procedure is PVI, which can be achieved in more than 95% of patients at the end of the procedure [2]. These results are obtained with similar success rates with radiofrequency ablation (RF), cryoablation, and laser ablation [2].

However, AF recurrence rates remain high and substantially unchanged in recent years [2]. AF recurrences are related to recovery of PV-LA conduction; according to some studies, in paroxysmal and persistent AF, the recovery of the PV-LA conduction is associated with high recurrence rates. Conversely, in paroxysmal AF patients, recurrences are extremely rare in patients who maintain permanent PVI [3, 4]. These concepts can be translated, at least partially, to persistent AF, in which the importance of the recovery of the PV-LA conduction has also been observed [3, 5].

It seems therefore essential to ensure, in the acute setting, the effectiveness and durability of the PVI, as this has a direct impact on the long-term outcome. When performing PVI, two main issues have to be addressed: first, how to discern whether permanent isolation (scar tissue) or a reversible injury (oedema) has been obtained?; second, how to know if acutely permanent isolation is going to predict absence of reconnection in the long term?

More than ten years ago, the use of adenosine in PVI was first described [6]. It was administered following initial PVI to unmask dormant pulmonary vein conduction (DC), reversible injury, and identify vein reconnection. Adenosine is now commonly used in clinical practice to assess DC with the assumption that additional ablation at sites of reconnection may improve long-term freedom from AF. The aim of this paper is to review the available literature addressing the role of adenosine testing and determine the impact of ablation at sites of PV reconnection on freedom from AF following PVI.

## 2. Mechanism of Adenosine

First described by Arentz et al. in 2004 [6], the infusion of adenosine after PVI was shown to be able to unmask incomplete lesions. After this observation, several studies found similar results but it was only in 2010 that Datino et al. [7] elegantly elucidated the mechanism of action of adenosine.

To understand adenosine's action, it is necessary to review the effect of RF energy on the LA myocardial cells (assuming the same net effect for other energy sources). After RF ablation, the cell membrane is damaged and is unable to maintain the resting potential, which is the ability to hyperpolarize the cell membrane compared to the extracellular space. This damage changes the resting potential to a level that is above the excitability threshold, making the cell unable to depolarize and conduct.

Potassium currents, activated during the recovery phase of the action potential (AP), contribute to the membrane hyperpolarization and are also damaged by RF lesions. Transient outward potassium currents (IK_ado_) are especially present in PV cells and in the LA-PV junction.

**Figure 1:**
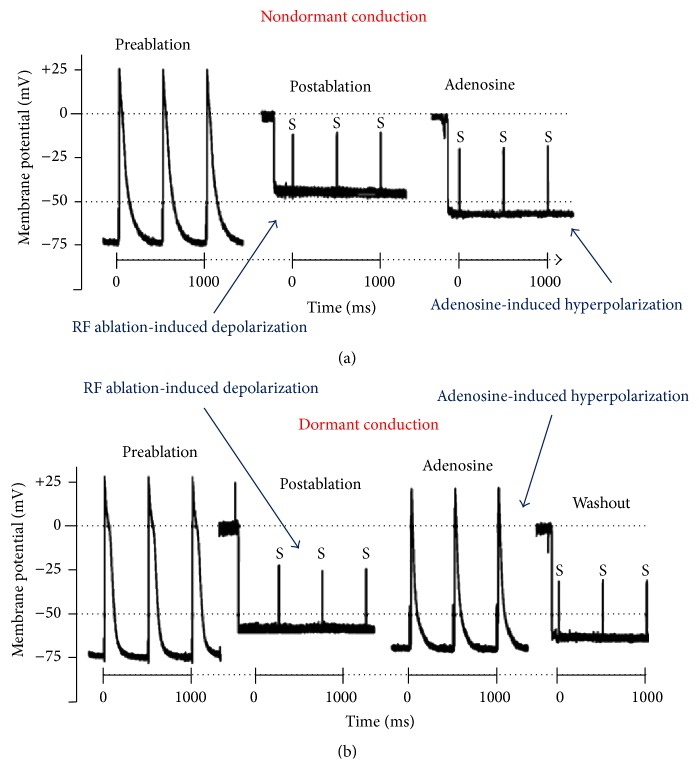
Microelectrode recordings before and after pulmonary vein isolation in a PV without dormant conduction (upper panel) and in a PV with dormant conduction (lower panel). S: stimulus artifacts with no response. Dotted line: excitability threshold at −50 mV (adapted from Datino et al. [7]).

Adenosine is able to increase these currents facilitating the membrane hyperpolarization and restoring the excitability threshold (Figure 1). Adenosine has a differential effect on PV cells and LA cells. It is able to shorten the action potential duration in both PV and LA cells but significantly hyperpolarizes the resting membrane potential and increases *dV*/*dt*_max⁡_ only in PV cells. After radiofrequency ablation, if the membrane damage is not complete and permanent, adenosine infusion can favour recovery of the resting potential and restore excitability. Those cells that have suffered complete and irreversible damage will not be able to respond to adenosine infusion and their membrane will remain depolarized and unexcitable.

Adenosine is also active on the Na channel of the PV cells by removing the voltage-dependent *I*_Na_ inactivation and increasing the *dV*/*dt*_max⁡_ (maximum velocity of phase 0 of the AP). An interesting study by Cheung et al. [8] noted that the PV reconnection occurs during the bradycardia phase of adenosine infusion, confirming the hyperpolarization mechanism underlying the adenosine effect.

The common final pathway of adenosine infusion is to recover excitability and conduction capacity of those cells with partial damage, thus unmasking reversible injury that could lead to recovery of PV-LA conduction and favour AF recurrences.

## 3. Studies Investigating the Use of Adenosine in PVI

Eleven studies addressing the use of adenosine in radiofrequency PVI were identified (Table 1). In total, 3775 patients were included in 4 retrospective studies (*n* = 845), 5 prospective nonrandomized studies (*n* = 283), and two prospective randomized studies (*n* = 2650): the ADVICE trial (Adenosine Following Pulmonary Vein Isolation to Target Dormant Conduction Elimination, *n* = 534) and the UNDER-ATP trial (UNmasking Dormant Electrical Reconduction by Adenosine Triphosphate, *n* = 2113), which will be analyzed separately [9–11].

These studies have mainly followed two algorithms to assess the utility of adenosine in PVI.

### 3.1. Adenosine Given versus Adenosine Not Given

**Figure 2:**
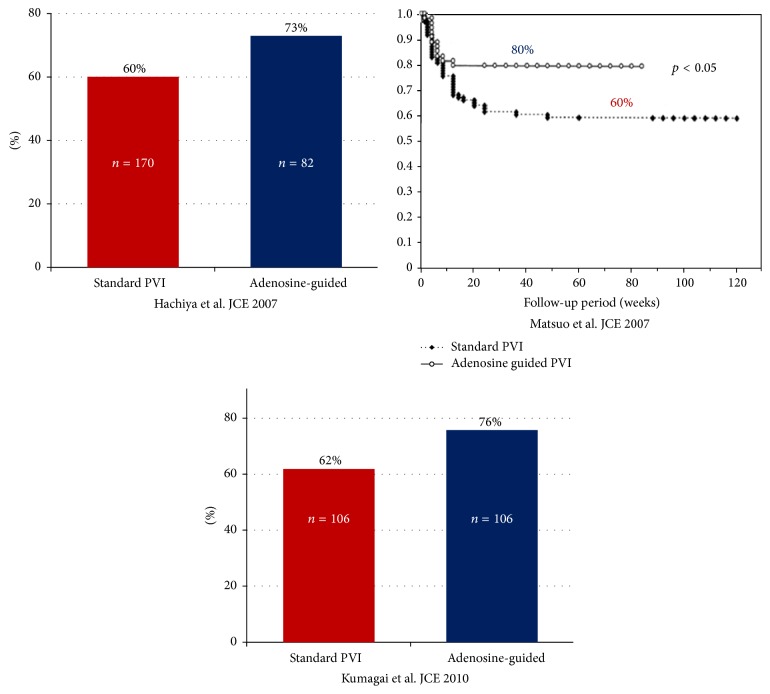
Freedom from AF in three nonrandomized retrospective studies comparing adenosine given versus adenosine not given [12–14]. PVI: pulmonary vein isolation.

Three retrospective studies addressed the impact of adenosine administration after PVI on freedom from AF [12–14] (Table 1). Each study compared a group of patients undergoing adenosine infusion after PVI (and additional ablation in case of DC) with a historical cohort from the same center of PVI patients not receiving adenosine. These studies included 612 patients, 242 of whom received adenosine after PVI. PV reconnection after adenosine infusion was observed in 190 PVs (31%) in 118 patients (49%). The time at which adenosine was given was 20 minutes after PVI in 1 study [13] and not specified in 2 studies [12, 14]. Additional ablation was performed at sites of reconnection until complete isolation of PVs after further adenosine testing. After an average follow-up of 14 months, cohorts of patients who had received routine adenosine administration after PVI showed better outcome with lower AF recurrence rate (Figure 2). The mean overall freedom from AF in patients given adenosine was 76% (73%–80%) versus 61% (60%–62%) in patients not tested.

### 3.2. Reconnection and Reablation versus No Reconnection

**Figure 3:**
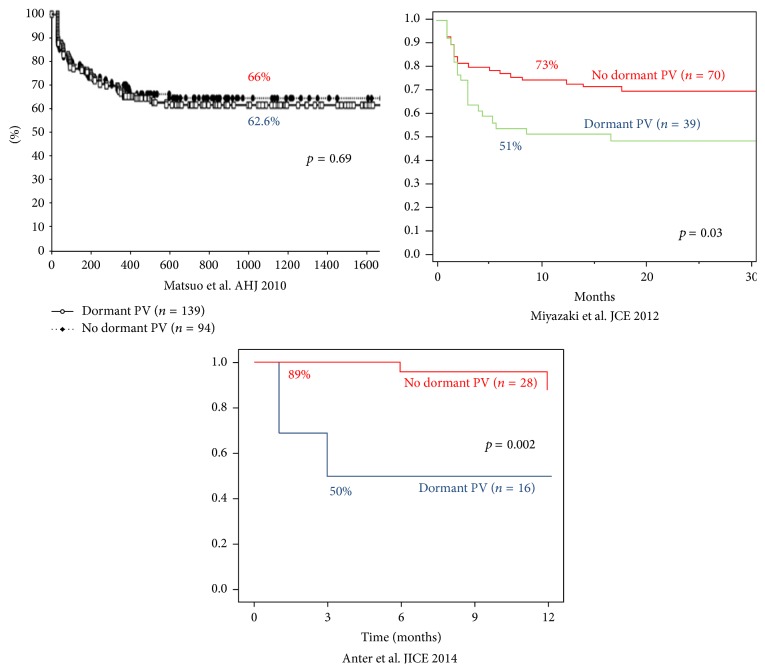
Freedom from AF in three nonrandomized studies comparing patients with adenosine induced reconnection and reablation (dormant PV) versus no reconnection [15, 17, 18]. PV: pulmonary vein.

Seven studies, 5 prospective, involved 598 patients divided in two cohorts: an adenosine infusion was used in all patients, and those with DC were reablated until complete isolation of PVs and were compared to patients without DC [6, 12, 15–19] (Table 1). In two studies (101 patients) reablation was not performed in cases of reconnection [6, 16]. 452 patients had paroxysmal AF (76%). The timing of adenosine administration was variable, ranging from immediately after PVI to 30 minutes after PVI. In total, patients with DC after adenosine testing were 282 (47%) and out of 1569 veins tested, 23.7% demonstrated DC (1 study did not specify the total number of veins tested [15]). After an average follow-up of 13 months, patients with DC and reablation did not show better outcome in terms of freedom from AF, but in fact they had an overall nonsignificant trend to worse outcome: 61% for patients with reconnection (38%–76%) and 71.5% in cases with no DC (44%–89%) [20] (Figure 3). Excluding those studies that did not perform further ablation in cases of DC [6, 16], the same outcome was observed (61% versus 72% AF freedom).

### 3.3. Interpretation of Study Results

Due to the many differences observed between these studies, it seems particularly difficult to definitively interpret the results presented. Most studies are retrospective with a historical cohort as the comparator, which involves limitations related to the period effect, the technological advances, and innovations. In addition, ablation techniques differ substantially between studies: segmental PVI was the technique of choice in the earliest studies while circumferential antral ablation or wide area encirclement was mostly used in more recently published ones. The differences in ablation techniques may influence the results in terms of AF recurrence and could introduce a bias when evaluating the role of adenosine. In some studies, the PVI was performed by antral ablation and electroanatomical mapping followed by careful mapping of the DC sites after adenosine administration [15, 18]. This technique allowed accurate mapping of the earliest adenosine induced PV activation and could help in guiding further ablation lesions. In comparison with purely anatomical PV encirclement, this electrophysiological approach may help in optimizing the adenosine test, perhaps resulting in better outcome in terms of AF recurrences.

Furthermore, adenosine dose and the presence of concurrent isoproterenol infusion show large variations across the studies, and not all of them are specified which was the endpoint of adenosine administration, with the presence of temporary heart block as only a surrogate goal. Finally, the wide range in timing of adenosine administration after PVI could be an important issue when evaluating early conduction recovery after ablation.

Despite these differences, the results seem to suggest that the presence of dormant PV conduction revealed by adenosine could be a marker for the absence of transmurality of ablation encircling lesions in a vein at especially high risk of reconnection. This could be secondary to anatomical challenges or poor catheter contact and adenosine may help to unmask the presence of ineffectively ablated areas [21]. In this setting, the use of the adenosine test, even if in retrospective studies, has appeared to improve outcomes compared to patients not tested.

On the other hand, it could be expected that further ablation at sites of reconnection should lead to higher freedom from AF recurrences but all studies analyzed failed to demonstrate this assumption.

Generally, in the studies described, the additional ablation is limited to the immediate site of PV reconnection and no further extension of the waiting period or more extensive reinforcement of the circumferential ablation line was performed. So the overall picture is that it is important to unmask veins at high risk but the usefulness of further ablation is yet to be determined.

### 3.4. The ADVICE Trial

The ADVICE trial is first prospective multicenter randomized study addressing the role of adenosine in PVI [9, 10]. The aim of the study was to determine whether an adenosine-guided ablation strategy improves the long-term efficacy of PVI for the treatment of paroxysmal AF. Of the 534 patients undergoing adenosine infusion, 284 (53%) had acute reconnection; 147 of them were randomized to receive no further ablation while 137 received additional RF pulses (over 95% success in eliminating DC). Out of the 250 patients without reconnection, 117 were randomized to an intense follow-up registry.

It is important to note that all patients underwent a 20-minute waiting period after PVI, and after that spontaneous vein reconnection (27% of patients, 9% of PVs) was eliminated and then adenosine was administered. Every vein was tested separately by means of a circular catheter inserted into the vein at the moment of the adenosine infusion, allowing the exact localization of the reconnection point.

**Figure 4:**
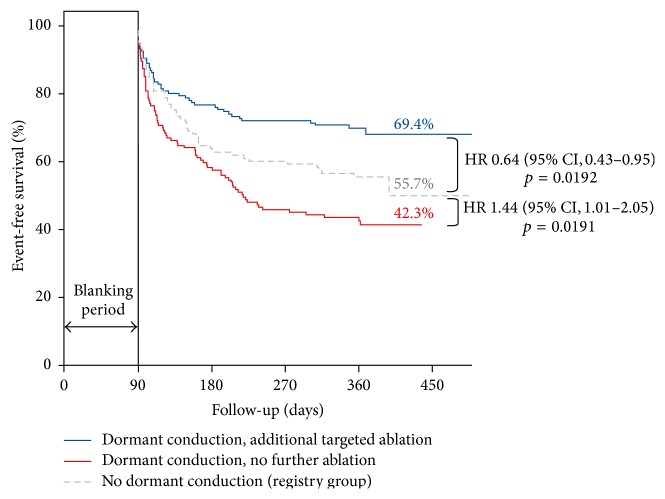
Freedom from symptomatic atrial tachyarrhythmia after a single ablation procedure in the ADVICE trial [9, 10].

The primary endpoint was the first documented symptomatic atrial tachyarrhythmia recurrence (AF, atrial flutter, and atrial tachycardia) after the blanking period. After 12 months of follow-up, patients showing DC and receiving additional ablation had a better outcome in terms of freedom from atrial tachyarrhythmias (69.4%) than patients with DC but without further ablation (42.3%, HR 0.44, *p* < 0.001) and also than those without reconnection (55.7%, HR 0.64, *p* = 0.019) included in the intense follow-up registry (Figure 4).

Need for repeated ablation during follow-up was higher in cases of acute reconnection without additional ablation (35%) compared to patients undergoing further ablation (20.4%, OR 0.48, *p* = 0.006). The authors concluded that DC is associated with increased risk of atrial tachyarrhythmia recurrence. Elimination of dormant pulmonary vein conduction reduces recurrent atrial tachyarrhythmias by >50%. They consider that these results support the routine use of adenosine for identification and elimination of DC during PVI procedures for paroxysmal AF.

The ADVICE trial adds important information on the role of adenosine infusion in PVI. On the one hand, it shows that patients with DC not receiving further ablation are extremely prone to AF recurrences. This result had been suggested to a point by the nonrandomized studies involving patients not tested with adenosine; the ADVICE trial confirms that speculation and shows the magnitude of the recurrence rate among those high risk patients. It clearly underlines the importance of looking for reconnection after an appropriate waiting time. Furthermore by clearly separating the effect of waiting time on spontaneous reconnection from the adenosine administration, the study may be able to evaluate the net effect of adenosine testing on the presence of DC.

The second important finding of the ADVICE trial is that further ablation of reconnected veins led to 12-month freedom from symptomatic atrial tachyarrhythmia after a single ablation procedure that was significantly higher than that in patients with DC not reablated. This seems to indicate that, after unmasking DC, it is useful to perform additional ablation to improve long-term outcome.

The third group of patients, those without DC, were intensively followed during 12 months and demonstrated higher atrial tachyarrhythmia recurrence rates than patients with DC and reablation. This finding may be considered quite surprising as this group of patients could be classified as being at low risk of vein reconnection, after a waiting period, ablation of spontaneous reconnection, and negative adenosine test.

As presented above, previous studies were unable to identify a benefit in further ablation, and on the contrary there was a trend, although not significant, towards a higher rate of recurrence compared to patients without reconnection.

One may speculate that negativity to adenosine test is not, per se, an indicator of low risk of recurrence but instead a response that leaves the door open to a possible reconnection, perhaps occurring not so early. In fact adenosine response is not an all-or-nothing phenomenon, but it is strictly related to the degree of cell damage, and its ability to restore the excitability threshold depends on the degree of depolarization the cell has suffered. In view of these considerations, the results of the ADVICE trial may suggest that patients without adenosine unmasked DC should be checked very carefully and perhaps, as discussed later, given more time during a waiting period.

### 3.5. The UNDER-ATP Trial

The UNDER-ATP trial is a recently published prospective multicenter randomized study addressing the role of adenosine in PVI [11].

The aim of the study was to determine whether an adenosine-guided ablation strategy improves the long-term efficacy of PVI for the treatment of paroxysmal, persistent (22.7%), and long lasting (10.1%) AF. Of the 2113 enrolled patients, 1001 were randomized to standard PVI and subsequent follow-up while 1112 underwent adenosine infusion at a fixed dose of 0.4 mg/Kg body weight after a variable waiting time following PVI (median time 43 min). After waiting time, 42% of patients in both groups had spontaneous reconnection that was targeted with further ablation. Thereafter, adenosine test was performed in the adenosine-guided PVI group, unmasking dormant conduction in 489 PVs among 307 patients (27.6%). Additional RF was delivered until dormant conduction was completely eliminated (98% of success). Almost 74% of patients were ablated with double circular catheter technique; consequently, PVs were tested with a single adenosine infusion for each pair of veins. A considerable number of patients received additional lines, specifically complex fractionated electrogram ablation (12%), mitral isthmus line (6.7%), roof line (18.2%), and superior vena cava isolation (13.9%).

The primary endpoint was recurrent atrial tachyarrhythmias at 1 year with the blanking period of 90 days after PVI.

After 12 months of follow-up, no differences were observed between groups, and 68.7% of patients in the adenosine-guided PVI group and 67.1% of patients in the conventional PVI group were free from the primary endpoint. Subgroup analysis showed no differences in terms of primary endpoint between patients with paroxysmal AF versus persistent/long standing and between patients with PVI alone versus PVI + additional lines.

The results of this study are clearly divergent from those obtained by the ADVICE trial. To understand these differences, it may be helpful to carefully analyze methodology and treatment approach. The ADVICE trial allowed a fixed waiting time of 20 minutes after completion of PVI and the dose of adenosine used depended on the ability to produce AV block or sinus pause. In the UNDER-ATP trial, the dose of adenosine was predetermined and the same for every patient, regardless of the effect produced. Moreover, there was no protocol regarding timing of adenosine administration, which was infused after the left atrial ablation was complete, resulting in a median time from PVI to adenosine more than double that of the ADVICE trial. On this basis, it is not surprising that in the UNDER-ATP trial the spontaneous reconnection was higher and the adenosine induced dormant conduction was lower than the ADVICE trial. Next, the ADVICE trial examined only patients with paroxysmal AF treated with PVI alone. In the UNDER-ATP trial, 32.8% of the patients had persistent or long standing AF and PVI procedure was often accompanied by additional linear lesions or complex electrogram ablation. This is remarkable since it is known that PVI in paroxysmal AF targets pulmonary vein triggers and adenosine test may help in assuring complete PVs isolation but it lacks any effect in case of additional linear lesions or non-PVs triggers. Moreover, patients with persistent or long standing AF develop more advanced disease with other driving mechanisms that may increase the risk of recurrence irrespective of the adenosine test results or the PVs reconnection.

There is another aspect to consider when analyzing the differences between the two studies. The UNDER-ATP trial used an anatomical approach to PVI by extensively encircling ipsilateral PVs mostly with the use of double circular catheters. The double circular catheter was also used for adenosine testing, preventing from accurate mapping of the DC site and tagging of the reconnection points before further RF delivery. Conversely, the ADVICE trial followed a different approach, based on the EP mapping of the DC sites for each vein that were tagged and targeted with further ablation.

These considerations may help in understanding the differences observed between both studies in order to extract concepts that can be useful in clinical practice. It can be suggested that adenosine test should probably be confined to patients with paroxysmal AF treated with PVI alone and that the accurate EP mapping of the DC site is a requisite for a reliable adenosine test.

#### 3.5.1. Other Energy Sources

The use of adenosine in PVI has also been studied in cases of ablation with other energy sources. Although with less amount of evidence, cryoablation and laser ablation already have some studies that have examined the role of adenosine (Table 2). There are 5 cryoablation studies, 3 of them with a control group [22–26], that analyzed the presence of DC after adenosine infusion. In cases of appearance of DC, additional applications were performed by cryoballoon or with a conventional cryothermal catheter until disappearance of DC after further adenosine infusion. The lack of homogeneity between these studies, as seen for RF ablation papers, prevents a reliable generalization of the results. The differences in the methodology used, the limited number of patients included, the presence of historical cohorts as a comparator, the varying dose of adenosine, and the presence or nonpresence of waiting time require extremely careful interpretation of the results. The overall picture is that the transmurality of the lesions is clearly related to positivity to adenosine: these studies revealed that the isolation of the veins with the first freeze, the time to isolation, and the nadir temperature are factors that identify veins being permanently isolated and less prone to DC and long-term recurrence. Two studies [23, 26] observed that freedom from AF recurrences was higher in the adenosine and reablation group compared to patients that did not receive adenosine. In one study, it has been observed that ablation with additional DC evaluation and treatment was found to independently reduce the risk of AF recurrence at follow-up [26]. Furthermore, the best results were obtained when the waiting time was combined with adenosine infusion. Finally, it can be noted (Table 2) that the percentage of patients with DC (and therefore the percentage of veins) seems less in cryoablation than in RF studies. The reason for this finding is unclear; however, it has been suggested that one of the strengths of cryoablation is the ability to create continuous and contiguous injuries, whose absence could be related to higher positivity to adenosine [29]. On the other hand, this finding could be related to a different time dependency of the adenosine response in cryoablation compared to RF, as it could be suggested by the similar success rates of both techniques in published studies in terms of freedom from AF. Probably the different mechanism of lesion formation implies a different time course of the adenosine response. Nevertheless, the absence of direct comparison and the paucity of evidence on this subject make every interpretation merely speculative.

Regarding laser ablation, evidence about the use of adenosine is more limited; one study [27] compared cryoablation with laser ablation and found higher rates of DC in laser ablation, but with the same AF recurrence rates after 1 year. Another study [28] observed that DC is associated with poor vein occlusion, longer duration of laser application, lower mean power of the applied laser energy, and higher overall number of 5.5 W applications; targeting veins with dormant conduction seems to improve rates of freedom from AF.

## 4. Waiting Time

Waiting time is a key variable when assessing PV reconnection after isolation. The mechanism of injury, based on the cell membrane damage and subsequent death, suggests that ensuring a permanent lesion requires some waiting time. How much time should be waited after PVI is a matter of discussion as there is a wide range of waiting times in the studies analyzed, and in the daily setting it could be difficult to allow more than 20 minutes after PVI. It has to be taken into account that the waiting time alone has a very important value because it allows those transient and nontransmural injuries to recover and restore LA-PV conduction.

The recovery of conduction is greater when increasing the waiting time and in some cases LA-PV conduction has been observed 60 minutes or more after ablation [30]. Cheema and colleagues [31] showed that patients with 60 minutes of waiting time had reconnection in >30% of the PVs and such patients, after additional ablation, had significantly higher freedom from AF than patients without any waiting time after PVI.

**Figure 5:**
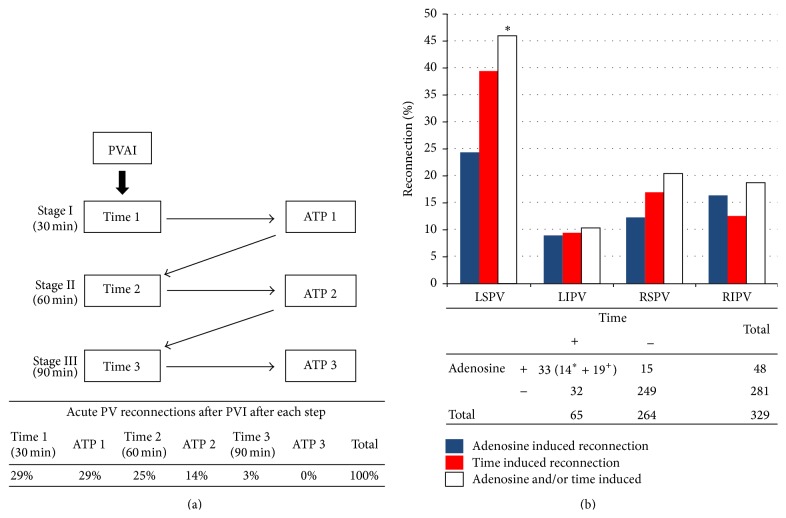
(a) Study algorithm and results from Yamane et al. [32]; PVAI: pulmonary vein isolation, ATP: Adenosine Triphosphate. (b) Incidence of reconnection in individual pulmonary veins mediated by adenosine and/or time, adapted from Jiang et al. [33] (see text for details).

Yamane and colleagues [32] combined the use of waiting time and adenosine: the study algorithm had 3 subsequent stages, each of them constituted by 30 minutes of waiting time and adenosine infusion (Figure 5(a)), followed by additional ablation at each step in case of reconnection. They proved that reconnection can be observed until 90 minutes after PVI and after 2 separated adenosine administrations. Thanks to this protocol, which obviously prolonged procedure times, they obtained 92% of freedom from AF after 1 year. These studies suggest that both waiting time and adenosine are useful when assessing permanent lesions after PVI but it remains unclear whether they provide the same information.

Jiang et al. [33] studied the relationship between waiting time and adenosine: immediately after PVI adenosine (20 mg) was infused, a waiting period of 30 minutes was allowed before assessment of reconnection (Figure 5(b)). Out of 329 PVs, 80 presented reconnection: 15 were adenosine positive but did not have permanent reconnection after the waiting time, while, out of 65 time-positive reconnections, 32 were adenosine-negative. They also noted that most of the PVs that reconnected with both adenosine-induction and waiting time after 30′ after PVI were found to be conducted at the same gap. This study concluded that the agreement between the two techniques is only moderate (*K* = 0.5) and that adenosine infusion may facilitate the process of time dependent reconnection.

In conclusion, it could be claimed that adenosine's effect may vary according to the degree of cell damage and membrane depolarization. In case of greater damage, adenosine may not be immediately able to restore the hyperpolarization required to reach the excitability threshold and it may be possible that only after enough waiting time its facilitating action could lead to the recovery of the LA-PV conduction. Therefore, the role of adenosine and waiting time can be considered as interdependent and it is advisable to use both techniques to ensure permanent injuries.

## 5. Prediction of Reconnection

As discussed above, adenosine seems quite useful to predict acute reconnection and possibly additional ablation may help in reducing the risk of AF recurrence. Nevertheless, it has still to be clarified if long-term PV reconnection, by far the most important cause of AF recurrence after PVI, can be predicted by adenosine induced dormant conduction.

Gula and coworkers [16] suggested that adenosine testing for the assessment of transient conduction recovery does not appear to predict recurrence of clinical AF. In 18 redo procedures, they observed 31 reconnected veins: 9 had been positive to adenosine and 22 negative at initial ablation. In this study, adenosine correctly predicted 13 out of 36 veins, resulting in positive predictive value (PPV) of 90% and negative predictive value (NPV) of 15%.

**Figure 6:**
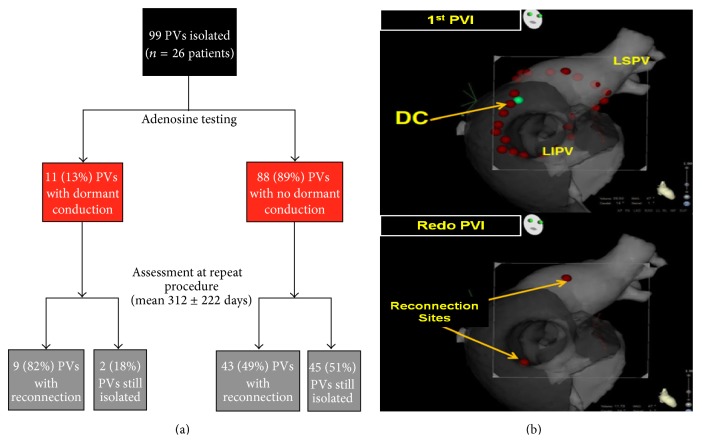
(a) Adenosine testing results and PV reconnection rate at redo procedure in Lin et al. [34]. (b) Example of redo procedure reconnection sites that totally differed from DC sites at first procedure [35]. DC: dormant conduction. PVI: pulmonary vein isolation (see text for details).

Lin et al. [34] described similar results in their study designed to address the ability of DC to predict AF recurrence and PV reconnection. In 26 redo procedures, they observed 53% rate of chronic PV reconnection (52 out of 99 PVs). DC had a PPV of 82% and NPV of 51% (Figure 6(a)). In these 2 studies, adenosine was shown to have good specificity and predictive value for future PV reconnection, especially if DC was left unablated [16]. Sensitivity was low and adenosine was unable to rule out chronic reconnection in case of absence of DC.

These studies analyzed each vein as a whole and did not focus on the precise spot of reconnection compared to the spot of DC at initial ablation procedure. In order to have reliable information about adenosine's ability to predict outcome, it appears to be extremely important to understand if a vein reconnects in the same spot with DC. Okishige and coworkers [35] performed a prospective study to assess the predictive value of the response to adenosine in terms of identifying the reconnection sites associated with AF recurrence. In their study, sites of DC were tagged using a three-dimensional mapping system and left unablated. DC was observed in 56 out of 91 patients (62%). After an average follow-up of 15 months, 62 patients were free from AF recurrences; 32 of them (52%) had DC during the first PVI. A second ablation procedure was performed in 29 patients (32%), all of them with DC during the first PVI.

Nineteen (66%) patients had reconnections sites that all differed from those of the DC sites, whilst only 3 patients (10%) had all reconnections sites identical to the DC sites. The remaining patients (24%) had reconnection sites that involved not only DC sites but also different sites (Figure 6(b)). The kappa test showed a poor agreement between the DC sites and reconnection sites in all PVs (*κ* = 0.157). The overall result is that the vast majority of the reconnection sites differed from the DC sites that were detected by an adenosine injection in the first session. Finally, the ADVICE trial showed that, in 110 redo procedures, the reconnection rate was 48% for PVs with DC and reablation (*n* = 40), 55% for PVs without DC (*n* = 229), and 88% for PVs with DC without reablation (*n* = 82).

In conclusion, there seems to be enough evidence to affirm that, despite a good specificity and PPV, the high number of false negative results (very low sensitivity) substantially decreases the diagnostic capacity of adenosine in terms of predicting long-term PV reconnection. These findings strengthen the idea that the overall benefit of adenosine testing and targeted ablation of DC is mostly related to the elimination of those lesions that appear to be nontransmural in the acute phase, with possible positive prognostic effect, according to the ADVICE trial results. On the contrary, the usefulness of the adenosine test as a predictor of chronic reconnection is likely to be modest at best.

## 6. Other Techniques

New techniques have been described in order to improve ability of identifying incomplete lesion after RF ablation. Among new technologies, the use of Pace-capture PVI could be useful in facilitating identification of residual gaps [36]. Andrade et al. compared 40 patients that received additional ablation in case of atrial capture at high output pacing on the ablation line with a group of patients with additional ablation in case of adenosine revealed DC. Rates of pace capture in the pace-capture group and rates of DC in the adenosine group were found to be similar but patients in the pace-capture group who underwent adenosine testing showed significant reduction in the incidence of DC [37].

Recently the use of contact force catheters has been introduced in RF ablation with the aim to obtain true permanent lesions more efficaciously than standard catheters [38]. It has been shown that the use of contact forces helps in improving AF ablation effectiveness [39] and may lead to better long-term results in terms of freedom from AF [40]. One study evaluated adenosine response with the use of contact force catheters and observed that the presence of DC is significantly reduced compared to standard catheters, suggesting that this could be related to better AF freedom in the long term [41].

## 7. Conclusion

This critical review tries to offer a complete overview on the role of adenosine in PVI based on the available literature.

Data presented suggests that adenosine is useful to unmask nontransmural lesions at high risk of reconnection. This means that further ablation at sites of DC is associated with improvement in freedom from AF after PVI. Therefore, based on these considerations, adenosine should be used routinely in clinical practice. Nevertheless, it is fundamental to keep in mind the limitations of the adenosine testing. In the acute phase, adenosine use should be accompanied by enough waiting time, as the adenosine response immediately after RF lesion may lack real value. Moreover, it has been demonstrated that adenosine is not able to predict all veins at risk of reconnection, mostly due to the very low negative predictive value, which means that veins without DC are not necessarily at low risk of reconnection.

Risks of PV reconnection and AF recurrence are related to the ability of creating reliable permanent scar. Among new technologies, contact force catheters improve lesion transmurality and significantly reduce the incidence of DC.

In conclusion, the objective of PVI should be trying to obtain complete and permanent lesions from the beginning, and the combination of adenosine, waiting time, and contact force assessment seems at the moment the best strategy to achieve this goal.

## Figures and Tables

**Table 1:** Radiofrequency ablation studies addressing adenosine use in PVI.

Study	*N*	Comparison groups	*n* (%)	*n* veins	pAF (%)	Waiting time (min)	Adenosine dose (mg)	Tested veins	Reconnected veins (%)	Follow-up (months)	AF-free (%)	*p*	Redo procedure	Reconnected veins in redo	
Adenosine given versus adenosine not given															

Hachiya et al. (2007) [12]^†^ Retrospective	252	Adenosine given Adenosine not given	82 170	164 —	62 (76) 135 (79)	— —	30 —	164 —	41 (25%) in 34 patients (41%) —	6.1 ± 3.3	60 (73%) 102 (60%)	0.04	—	—	

Matsuo et al. (2007) [13]^†^ Retrospective	148	Adenosine given Adenosine not given	54 94	224 —	36 (67) 60 (64)	20 —	20 —	224 —	59 (26%) in 30 patients (56%) —	19.9 ± 6	43 (80%) 56 (60%)	<0.05	9 (17%) 36 (38%)	DC+ DC−	8/14 (57%) 12/22 (55%)

Kumagai et al. (2010) [14]^†^ Retrospective	212	Adenosine given Adenosine not given	106 106	216 —	94 (89) 86 (81)	— —	10 —	216 —	90 (42%) in 54 patients (51%) —	16 ± 5 16 ± 7	81 (76%) 66 (62%)	0.03	11 (10%) 10 (9.4%)	— —	

Kobori et al. (2015) [11]^*∗*^ Randomized Prospective	2113	Adenosine given Adenosine not given	1112 1001	—	1420 (67%)	43 —	0.4 mg/kg —	— —	307 in 1112 patients (27.6%) —	12	68.7% 67.1%	0.25	—	—	

Reconnection versus no reconnection															

Tritto et al. (2004) [19] Prospective	29	Reconnection No reconnection	16 (55) 13 (45)	74	21 (72)	10	12	62	22 (35%) 0	6.3 ± 2.4	11 (69%) 9 (69%)	1	6	DC+ DC−	6/8 (75%) 4/5 (80%)

Arentz et al. (2004) [6]^¶^ Prospective	29	Reconnection No reconnection	13 (45) 16 (55)	83	20 (69)	0	12–18	53	13 (24%) 0	12	5 (38%) 7 (44%)	1	14	DC+ DC−	5/7 (71%) 7/20 (35%)

Hachiya et al. (2007) [12]^*∗*^ Retrospective	82	Reconnection No reconnection	34 (41) 48 (59)	164	62 (76)	0	30	164	41 (25%) 0	6.1 ± 3.3	23 (68%) 37 (77%)	—	—	—	

Matsuo et al. (2010) [17] Retrospective	233	Reconnection No reconnection	139 (60) 94 (40)	930	144 (62)	20	20	928	225 0	29 ± 13	87 (62.6%) 62 (66%)	0.69	43 (31%) 28 (30%)	DC+ DC−	42/78 (54%) 119/202 (59%)

Gula et al. (2011) [16]^*∗*¶^ Prospective	72	Reconnection No reconnection	25 (35) 47 (65)	50 94	25 (100) 47 (100)	30	12	50 94	29 (58%) 0	12	19 (76%) 35 (74%)	1	6 (24%) 12 (26%)	DC+ DC−	9/10 (90%) 22/26 (85%)

Miyazaki et al. (2012) [18]^*∗*^ Prospective	109	Reconnection No reconnection	39 (36) 70 (64)	78 140	39 (100) 70 (100)	0	40	78 140	42 (54%) 0	12	20 (51%) 51 (73%)	0.03	10 (26%) 22 (31%)	DC+ DC−	6/10 (60%) 27/54 (50%)

Anter et al. (2014) [15] Prospective	44	Reconnection No reconnection	16 (36) 28 (64)	—	8 (50) 16 (57)	30	12–48	—	26 0	12	8 (50%) 25 (89%)	0.009	3 (7%)	— —	

Macle et al. (2015) [10]^#^ Randomized Prospective	401	Reconnection and no ablation Reconnection and ablation No reconnection	147 (28) 137 (26) 117^‡^	— — —	147 (100) 137 (100) 117 (100)	20	12–18	2085	— — 0	12	42.3% 69.4% 55.7%	<0.001 0.019	110	88% 48% 55%	

^*∗*^Veins considered and tested per pair; ^¶^no further ablation in case of dormant conduction; ^†^historical cohort as comparator; ^‡^out of 250 patients without dormant conduction 117 were randomized to intense follow-up registry. ^#^This study specified the freedom from any atrial tachycardia as the primary endpoint. DC+: veins with dormant conduction at first procedure; DC−: veins without dormant conduction at first procedure; pAF: paroxysmal atrial fibrillation.

**Table 2:** Cryoablation and laser ablation studies addressing adenosine use in PVI.

Study	*N*	Comparison groups	*n*	*n* veins	Energy	pAF	Waiting time (minutes)	Adenosine dose (mg)	Reconnected patients (%)	Reconnected veins (%)	Follow-up (months)	AF freedom (%)	*p*	Recurrence in patients with DC (%)
Chierchia et al. 2009 [22]	39	—	—	149	Cryo	100%	15	20	5 (13%)	7 (4.6%)	6	77%	—	2/5 (40%)

Van Belle et al. 2012 [23]	99	A given	34	132	Cryo	100%	0	25	7 (21%)	9 (8%)	17 ± 5	68%	0.04	—
		A not given	65	—	Cryo	100%	—	—	—	—	17 ± 5	46%		—

Ciconte et al. 2014 [24]	50	A given	—	200	Cryo	82%	30	18–30	6 (12%)	8 (4%)	7 ± 1.7	86%	—	0 (0%)

Kumar et al. 2015 [25]	90	A given	45	179	Cryo	79	30	15 ± 3	—	8 (4.5%)	13 ± 1	84%	NS	0 (0%)
		A not given	45	179	Cryo		—		—	—	12 ± 2	79%		—

Compier et al. 2015 [26]	98	A given	36	143	Cryo	86%	30	17 ± 5	15 (42%)	20 (14%)	12 ± 1	83%	0.02	—
		A not given (historical cohort)	62	—	Cryo	90%	—	—	—	—	11 ± 1	60%		—

Kumar et al. 2014 [27]	60	A given	40	151	Cryo	87.5%	30	12 ± 3	4 (2.5%)	4 (5%)	11 ± 3	85%	NS	—
		A given	20	80	Laser	90%	30	12 ± 3	7 (35%)	11 (13.8%)	9 ± 2	85%		—

Üçer et al. 2015 [28]	26	A given	—	102	Laser	100%	30	18	5 (20%)	6 (6.7%)	6	81%	—	2/5 (40%)

A: adenosine; AF: atrial fibrillation; DC: dormant conduction; pAF: paroxysmal atrial fibrillation.
